# Stress-Inducible Expression of an F-box Gene *TaFBA1* from Wheat Enhanced the Drought Tolerance in Transgenic Tobacco Plants without Impacting Growth and Development

**DOI:** 10.3389/fpls.2016.01295

**Published:** 2016-09-05

**Authors:** Xiangzhu Kong, Shumei Zhou, Suhong Yin, Zhongxian Zhao, Yangyang Han, Wei Wang

**Affiliations:** State Key Laboratory of Crop Biology, Shandong Key Laboratory of Crop Biology, College of Life Sciences, Shandong Agricultural UniversityTai’an, China

**Keywords:** F-box, *RD29A* promoter, drought stress, water retention, antioxidative compete ability

## Abstract

E3 ligase plays an important role in the response to many environment stresses in plants. In our previous study, constitutive overexpression of an F-box protein gene *TaFBA1* driven by *35S* promoter improved the drought tolerance in transgenic tobacco plants, but the growth and development in transgenic plants was altered in normal conditions. In this study, we used stress-inducible promoter *RD29A* instead of *35S* promoter, as a results, the stress-inducible transgenic tobacco plants exhibit a similar phenotype with wild type (WT) plants. However, the drought tolerance of the transgenic plants with stress-inducible expressed *TaFBA1* was enhanced. The improved drought tolerance of transgenic plants was indicated by their higher seed germination rate and survival rate, greater biomass and photosynthesis than those of WT under water stress, which may be related to their greater water retention capability and osmotic adjustment. Moreover, the transgenic plants accumulated less reactive oxygen species, kept lower MDA content and membrane leakage under water stress, which may be related to their higher levels of antioxidant enzyme activity and upregulated gene expression of some antioxidant enzymes. These results suggest that stress induced expression of *TaFBA1* confers drought tolerance via the improved water retention and antioxidative compete ability. Meanwhile, this stress-inducible expression strategy by *RD29A* promoter can minimize the unexpectable effects by *35S* constitutive promoter on phenotypes of the transgenic plants.

## Introduction

Abiotic stresses are the major limiting factors in plant growth and development, and can greatly affect crop production. Drought is one of the most common forms among abiotic stresses. Plants can response and adapt these stresses by many kinds of ways. For example, many genes may be up- or down- regulated to maintain the growth of plants under drought stress (Farooq et al., 2009). Meanwhile, lots of drought-related proteins accumulate to protect plants from the damage of deficit stress conditions (Bu et al., 2014). Although the responses of plants to drought are relatively widely considered, the molecular mechanisms of plant adaptation to drought are still fragmentary.

The ubiquitin-26S proteasome system (UPS) plays an important role in the resistance of plants to abiotic stress by affecting the stability of the cellular proteins (Perales et al., 2008; Stone, 2014). In the UPS, E3 acts as a central component in the ubiquitination process, which confirms the specificity to the target proteins (Dill et al., 2004). E3 ligase is a far more diverse group in plants. Among the E3 ligases, the SCF complex, named after the three key components Skp1, Cullin/CDC53 and F-box proteins, is essential in plants (Ciaffi et al., 2005). F-box protein, as a major subunit of the SCF complex, can bind to Skp1 through an F-box motif at the N-terminus of the protein, which consists of a 40–50 amino acid motif. The F-box protein recruits the targets via its C-terminus protein-protein interaction specificity to SCF E3. F-box proteins are involved in the response to biotic and abiotic stresses. *DOR* encodes an F-box protein in *Arabidopsis*, which functions as an inhibitor of ABA-induced stomatal closure under drought stress (Zhang et al., 2008). Overexpression of *OsDRF1*, a rice defense-related F-box protein gene, results in enhanced disease resistance against tomato mosaic virus (ToMV) and *Pseudomonas syringae* pv. *Tabaci*, and strengthens the sensibility to ABA in transgenic tobacco plants. *TdRF1* is a durum wheat nuclear ubiquitin ligase, which can respond to cold and drought stress, and its homologous gene *WVIP2* can negatively regulate the drought stress in *Triticum durum* (Guerra et al., 2012).

Drought stress may cause various adverse effects on plant growth and development, such as dwarf plant, smaller leaf area and decreased biomass. The maintenance of a high photosynthesis rate is a key factor for maintaining crop yields under stress conditions. Photosynthesis is sensitive to drought stress because water deficit results in the closing of stomata and decreases the internal leaf CO_2_ concentration (Dubey, 2005). Stress-induced changes are frequently related to an increase in membrane permeability, affecting membrane integrity and cell compartmentation under stress conditions. Reactive oxygen species (ROS)-mediated membrane injury involved in the membrane permeability during drought stress (Moore and Roberts, 1998).

In our previous study, we cloned an F-box protein gene from wheat (Zhou et al., 2014). The transgenic plants with overexpressed *TaFBA1* under the control of the constitutive *35S CaMV* promoter displayed a changed phenotype in growth and development under normal conditions (Zhou et al., 2014). In the present study, the stress-inducible *RD29A* promoter was used instead of *35S* promoter for *TaFBA1* overexpression to minimize the effects on plant growth and development. Encouragingly, *RD29A::TaFBA1* transgenic plants exhibited enhanced drought tolerance while avoiding the variation in the development process compared with *35S::TaFBA1* plants. We investigated the probable mechanism underlying the enhanced drought tolerance in the transgenic tobacco plants.

## Materials and Methods

### Ethics Statement

This work did not involve endangered or protected species. We abided by the statement of ethical standards for submitted manuscripts, and the manuscript does not describe experiments involving human subjects or animals.

### Plant Materials, Growth Conditions, and Stress Conditions

Transgenic tobacco (*Nicotiana tabacum*) lines containing the *RD29A::TaFBA1* vector (RF-3, RF-4, RF-9), *35S::TaFBA1* vector (T3, T8) and wild type (WT) were used in this study. *TaFBA1*, an F-box gene, was isolated from wheat (*Triticum aestivum* L.; Zhou et al., 2014).

The tobacco seeds were sown in pots (8 cm × 10 cm) containing vermiculite soaked with half-strength Hoagland nutrient solution in a growth chamber at 25°C with a 16/8 h (light/dark) cycle (300–400 μmol photons m^-2^ s^-2^) and relative humidity of 75–80%.

For water stress treatment to seed germination, the transgenic and WT seeds were sown in a solution containing 0, 10 or 20% PEG6000 (mass to volume ratio). For drought stress at the seedling stage, the transgenic and WT plants were germinated and grown under control growth conditions, and the water was withheld for a week to allow drought stress to develop. Then, these plants were rewatered and their growth was monitored after 3 days from rewatering.

For methyl viologen (MV) treatment, the transgenic and WT plants were sprayed with MV solution (100 μM) every 6 h for three times and the phenotypic change was observed after 24 h.

### RNA Extraction and cDNA Synthesis

Total RNA was extracted from the tobacco leaves with the Trizol reagent (TaKaRa, Japan) according to the manufactures, protocol and was treated with DNaseI (RNase-free, Promega). The total RNA was subjected to first-strand cDNA synthesis with the RevertAid First Strand cDNA Synthesis Kit (Fermentas, USA) according to the manufacturer’s protocol.

### Gene Expression Analysis by Quantitative Real-Time RT-PCR

*TaFBA1* expression was followed by the presence of a 222-bp fragment amplified with the primers QFBA1 and QFBA2. The Actin cDNA was used as a control. PCR was carried out in a final volume of 20 μl containing 1× SYBR Green PCR MASTER Mix (TIANGEN, China), 500 nM each primers and 120 fifth of the RT reaction. Quantitative analysis was performed using the Bio Rad CFX Manager system with PCR conditions of 94°C for 20 s, 61°C for 30 s and 68°C for 35 s for 40 cycles. The absence of primer–dimer formation was confirmed using single and no-primer controls. Each sample was performed in triplicate using relative quantification analysis. Some primers of antioxidative-related genes in tobacco were given in **Table 1**.

**Table 1:** Primer sequences were used in the article.

Name	Primer sequence (5′–3′)	Length (bp)
Ntactin-F	CATTGGCGCTGAGAGATTCC	20
Ntactin-R	GCAGCTTCCATTCCGATCA	19
NtSOD-F	GACGGACCTTAGCAACAGG	19
NtSOD-R	CTGTAAGTAGTATGCATGTTC	21
NtRbohD-F	ACCAGCACTGACCAAAGAA	19
NtRbohD-R	TAGCATCACAACCACAACTA	20
NtCAT1-F	TGGATCTCATACTGGTCTCA	20
NtCAT1-R	TTCCATTGTTTCAGTCATTCA	21
NtGPX-F	GGTTTGCACTCGCTTCAAG	19
NtGPX-R	AGTAGTGGCAAAACAGGAAG	20
NtAPX1-F	GAGAAATATGCTGCGGATGA	20
NtAPX1-R	CGTCTAATAACAGCTGCCAA	20
NtAPX2-F	GACAACTCATACTTTACGGA	20
NtAPX2-R	CTTCAGCAAATCCCAACTCA	20
QFBA1	AGCAGCAGAACAAGCCTGACCA	22
QFBA2	ACGTGACGTTGGACAGCCTTTG	22

### Western Blot Analysis

Total protein was extracted from tobacco leaves in protein extraction buffer (100 mM Tris-HCl, pH 8.0, 1% Polyvinylpyrrolidone, 10 mM β-mercaptoethanol, 1 mM EDTA-Na_2_, 0.2 M sucrose) as described previously (Lee et al., 2007). Samples were centrifuged for 10 min at 4°C, and supernatant containing soluble protein was harvested. Protein content was determined by the dye-binding assay according to Bradford (1976). Proteins were separated with SDS-PAGE on a 12% gradient gel and were electrophoretically transferred to polyvinylidene fluoride (PVDF) membranes (Millipore, Billerica, MA, USA). PVDF membranes were blocked for 2 h at room temperature in blocking buffer [11.25 mL Calf serum, 2.25 g Bovine Serum Albumin, and 63.75 mL phosphate buffered sodium (PBS)]. Next, membranes were incubated overnight at 4°C with TaFBA1 antibody diluted 1:1500 in blocking buffer. The coding region of *TaFBA1* was integrated into the Pet-30a (+) vector. Expression and purification of the recombinant TaFBA1 protein was processed using Ni-NTA agarose system. White mice were immunized by the purified recombinant protein to prepare antiserum, which was then purified to obtain the primary antibody aiming at TaFBA1 (Zhou et al., 2014). Membranes incubated with TaFBA1 antibody were washed three times for 15 min with PBS. Then, membranes were incubated for 3 h at room temperature with goat anti-mouse IgG, horseradish peroxidase conjugate (Santa Cruz Biotechnology, Santa Cruz, CA, USA) diluted 1:5000 in blocking buffer. Next, membranes were washed three times 15 min with PBS. Proteins were detected using chromogenic reagent (0.6 mL methanol, 3 μL H_2_O_2_, 1.5 mg 4-chloro-1-naphthol, and 5 mL PBS).

### Germination Rate Assay

Seeds from WT and *RD29A::TaFBA1* transgenic lines were sown on filter paper containing 0, 10 or 20% PEG-6000 and cultured in an incubator(16 h light/8 h dark, 75% humidity, 25°C). Germination rates were evaluated after 10 days.

### Measurement of Photosynthetic Gas Exchange and Chlorophyll Content

The adult tobacco plants were used to estimate the photosynthetic rate (Pn) with a portable photosynthetic system (CIRAS-2, PP Systems, Hitchin, UK). The measurements were carried out under the condition of a CO_2_ concentration of 360 μl l^-1^, PFD of 800 μmol m^-2^ s^-1^ and relative humidity of 60–70% and the temperature inside the leaf chamber was 25°C. Before the assay, all tobacco plants were treated at 25°C, 100 μmol m^-2^ s^-1^ PDF for at least 30 min to induce the stomata open, and then lighted at 800 μmol m^-2^ s^-1^ PDF for 15 min to be acclimated.

The chlorophyll content assay was carried out according to Yang et al. (2009).

### Measurement of Water Loss, Relative Water Content, and Water Potential

Measurement of water loss was carried out as Cheong et al. (2007). Relative water content (RWC) was calculated using the following equation (FW–DW)/(TW–DW) × 100%. Meanwhile, FW is the fresh weight, TW is the saturated weight after soaking the samples in water for at least 12 h and DW is the dry weight after the shaping at 105°C for 15 min and then drying samples at 65°C for 24 h. Water potential was measured with PSψPRO^TM^ water potential indicator (Wescor, USA) before and after drought treatment. Leaf disks from the third leaf were collected, immediately transferred to the chamber (C-52) which was equilibrated for 30 min before measurement. A stable instrument reading was obtained at that time.

### Determining of H_2_O_2_ content and O_2_^•-^ Production Rate

H_2_O_2_ content and O_2_^•-^ production rate was measured according to Li A.X. et al. (2014).

### Histochemical ROS Staining

H_2_O_2_ and O_2_^•-^ were detected by the 3,3′-diaminobenzidine (DAB) and nitrotetrazolium blue chloride (NBT) staining methods (Scarpeci et al., 2008; Zong et al., 2009). The seedlings were soaked in 5 mg/ml DAB at pH 3.8 for 20 h and 0.5 mg/ml NBT for 20 h in the dark condition to detect H_2_O_2_ and O_2_^•-^, respectively. Then the seedlings were subsequently decolorized by boiling in ethanol (96%) for 10 min. After cooling, the seedlings were extracted at room temperature with 60% glycerol and photographed.

### Determining of Malondialdehyde (MDA) Content and Electrolyte Leakage

Oxidative damage to lipids was estimates by measuring the content of MDA according to Quan et al. (2004). Electrolyte leakage was determined as described in Li A.X. et al. (2014).

### Extraction and Assay of Antioxidant Enzyme Activity

Tobacco seedlings treated with drought stress were used for the determining of superoxide dismutase (SOD, EC 1.15.1.1), catalase (CAT, EC 1.11.1.6), and peroxidase (POD, EC 1.11.1.7) enzyme activities as described previously (Li A.X. et al., 2014). The glutathione reductase (GR), dehydroascorbate reductase (DHAR), and monodehydroascorbate reductase (MDHAR) activities were determined according to Li et al. (2011). Enzyme activity assays were carried out in a UV-vis spectrophotometer (UV-2550, Shimadzu, Japan) at 25°C. The protein concentration of each enzyme extracts was determined according to the method of Bradford (1976).

### Statistical Analysis

All experiments were repeated at least three times. Statistical analysis was conducted using the procedures of SPSS, and statistical significance was tested at a probability level of 0.01 and 0.05.

## Results

### Generation and Identification of *RD29A::TaFBA1* Transgenic Tobacco Plants

**FIGURE 1:**
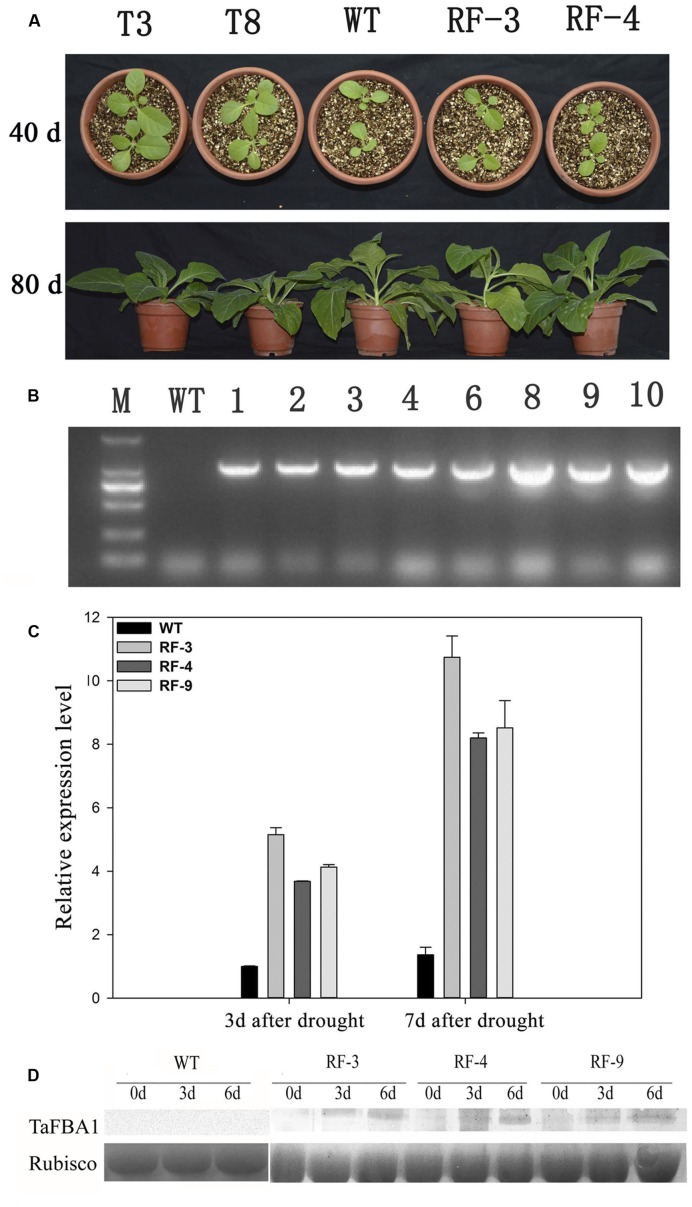
**Identification of RD29A::TaFBA1 transgenic tobacco lines. (A)** Phenotype of 35S::TaFBA1 (T3, T8), RD29A::TaFBA1 (RF-3, RF-4) and WT plants at 40, 80 days after sowing. **(B)** Identification of RD29A::TaFBA1 transgenic tobacco lines by genome PCR. WT, wild-type; M, DNA marker. **(C)** Identification of RD29A::TaFBA1 transgenic plants by real-time RT-PCR. Three independent transgenic lines (RF-3, 4, and 9) were used. **(D)** TaFBA1 protein abundance in transgenic plants in different drought conditions, as revealed by western blot analysis, rubisco large subunit was used as a loading control.

In our previously study, the full-length cDNA of *TaFBA1* gene was obtained from wheat (Zhou et al., 2014). Overexpression of *TaFBA1* in transgenic tobacco led to an altered phenotype compared with WT under normal condition (**Figure 1A**). To eliminate the phenotypical variation, we cloned a water deficit stress-inducible *RD29A* promoter from *Arabidopsis*, created a construct containing *RD29A::TaFBA1*, and transformed this construct into tobacco plants. *TaFBA1* was successfully integrated into the tobacco genome (**Figure 1B**). Homozygous progeny of three transgenic lines *RD29A::TaFBA1* (RF-3, RF-4, RF-9), *35S::TaFBA1*(T3, T8) and WT tobacco plants were used. Stress-inducible *RD29A* promoter was induced by drought stress. Three-month-old plants were grown without watering for drought stress. *TaFBA1* expression driven by *RD29A* promoter was increased during drought stress treatment (**Figure 1C**). Furthermore, the expression of *TaFBA1* at the protein level was confirmed by western blot analysis and the abundance of the TaFBA1 protein increased during drought stress treatment (**Figure 1D**), while the protein of WT was of no significant variety, which is similar to the result in **Figure 1C**.

### The Drought Tolerance of the Transgenic Tobacco Plants during Seed Germination and Seedling Growth

**FIGURE 2:**
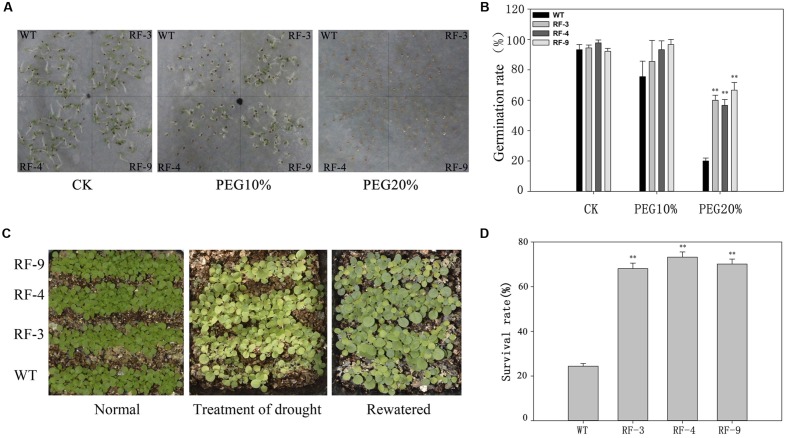
**The drought tolerance of transgenic tobacco seedlings with drought-inducible expression of *TaFBA1* and WT. (A)** Seed germination on filter paper containing 0 (CK), 15 and 20% PEG6000. **(B)** Germination rate (%) of RD29A::TaFBA1 and WT seeds in **(A)**. Germination rate was scored every day and the final result at 7 days after germination was presented. **(C,D)** Survival rates of transgenic and WT plants after withholding water for 10 days (Treatment of drought) and then rehydration for 3 days (Rewatered). The survival rate was scored according to the rewatered plants. The experiments were repeated three times. Each column represents the average of at least three replicates ± standard error (SE). “^∗^” and “^∗∗^” indicates significant differences compared with WT at *P* < 0.05 and *P* < 0.01.

Tobacco seeds of both WT and transgenic plants were sown in a solution containing 0, 10 or 20% PEG6000 (**Figure 2A**). Under normal conditions, WT and transgenic plants had the similar germination rates. But the germination rate of transgenic plant seeds was higher than that of WT under stress conditions. In the presence of 10 and 20% PEG6000, the germination rates of transgenic plants were approximately 90 and 60%, while those of WT were 80 and 20% (**Figure 2B**).

Wild type and transgenic lines were grown on soil for 2 weeks and then withdrawn water for 7 days. After the drought stress, we rewatered the plants for 3 days, and the survival rates were recorded (**Figure 2C**). The results showed that only 24.4% of WT were recovered from drought conditions after rewatering, while more than 70% of the transgenic plants survived (**Figure 2D**). These data suggest that the transgenic plants are less sensitive to drought stress compared with WT during seed germination and seedling stage.

### The Response of Growth and Photosynthesis of the Adult Transgenic Plants to Drought Stress

**FIGURE 3:**
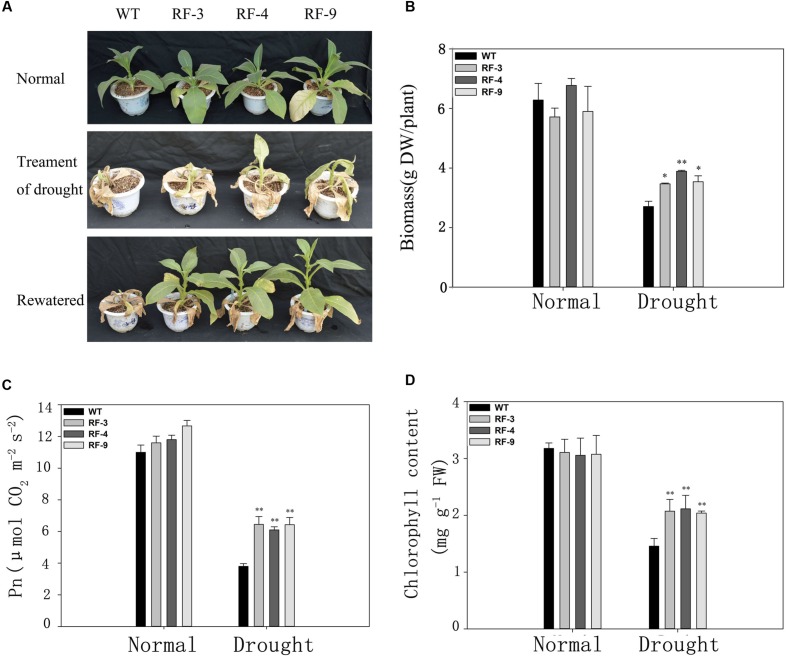
**Physiological characteristics of adult transgenic and WT plants in response to drought stress. (A)** Phenotype of transgenic and WT plants after withholding water for 15 days (Treatment of drought) and then rehydration for 3 days (Rewatered) in the greenhouse. **(B)** Overground biomass of each individual plant. Plants were cultivated in soil for 75 days. DW, dry weight. **(C)** Net photosynthetic rate (Pn). **(D)** The chlorophyll content of all lines after drought treatment. Each column represents the average of at least three replicates ± standard error (SE). “^∗^” and “^∗∗^” indicates significant differences compared with WT at *P* < 0.05 and *P* < 0.01.

Three-month-old plants were grown without watering for 15 days, and then rewatered for 3 days. The results showed that most of WT plants did not recover, while all three transgenic lines recovered and showed a vigorous growth status (**Figure 3A**). The biomass of transgenic plants was higher than these of WT (**Figure 3B**) when stressed for 15 days.

We also compared the photosynthetic indexes in the transgenic plants and WT under normal and drought conditions. Under normal condition, the photosynthetic rate (Pn) of transgenic tobacco lines was similar to that of WT. When suffering drought stress, Pn of transgenic plants was significantly higher than that of WT (**Figure 3C**), which is consistent with the phenotypic change (**Figure 3A**) and biomass (**Figure 3B**). The responses of chlorophyll content to drought stress in the transngenic lines and WT were consistent with photosynthetic rate (**Figure 3D**). The results in **Figures 2** and **3** suggest that the *RD29A::TaFBA1* transgenic tobacco plants had greater drought tolerance than WT plants.

### The *RD29A::TaFBA1* Transgenic Plants Display a High Water Retention Capability

**FIGURE 4:**
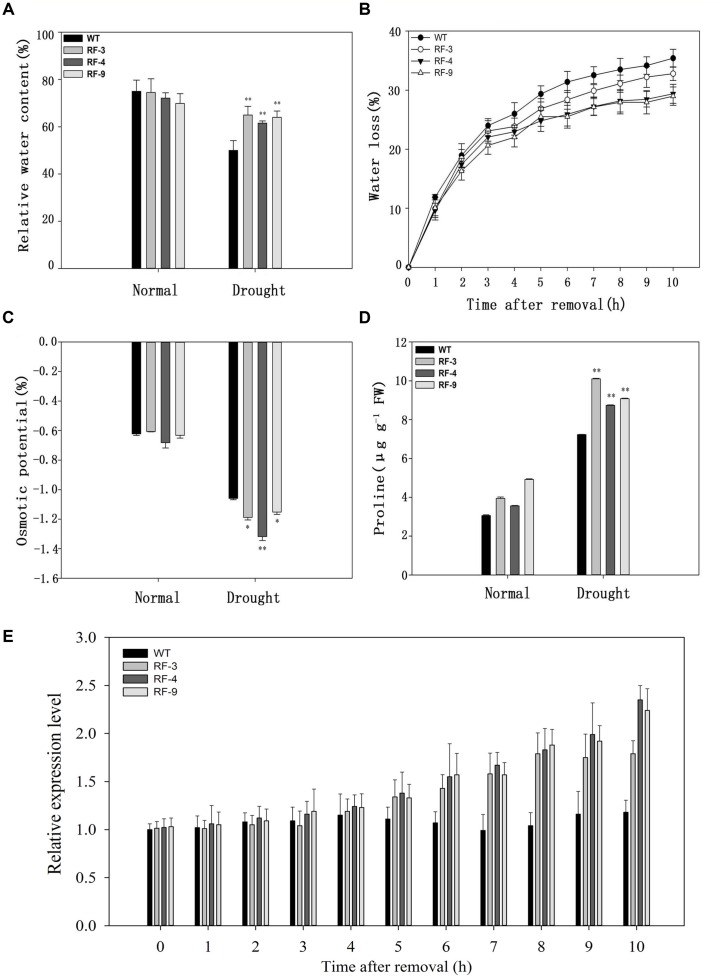
**The response of relative water content (RWC), water loss, osmotic potential, proline content and expression of *TaFBA1* in WT and transgenic plants. (A)** RWC of tobacco leaves after drought stress. **(B)** Kinetics of water loss from the leaves of 50-day-old tobacco plants. **(C)** Osmotic potential of tobacco leaves. **(D)** Proline content in WT and transgenic plant leaves. **(E)** Relative expression level of *TaFBA1* from the leaves of 50-day-old tobacco plants after removal. Each column represents the average of at least three replicates ± standard error (SE). “^∗^” and “^∗∗^” indicates significant differences compared with WT at *P* < 0.05 and *P* < 0.01.

We investigated the transpiration water loss and RWC in the transgenic plants and WT. The data in **Figure 4A** showed that there was no difference in RWC between WT and transgenic plants without stress, while the RWC level of transgenic plants was higher than that of WT when suffering drought stress. As shown in **Figure 4B**, the detached leaves of transgenic plants lost water more slowly than did those of WT. We also detected the relative expression level of *TaFBA1* in the transgenic plants and WT under normal and water loss conditions. The expression of *TaFBA1* was up-regulated in the transgenic plants under water loss conditions (**Figure 4E**).

In addition, transgenic plants exhibited lower osmotic potential than WT plants under water deficient conditions (**Figure 4C**). As transgenic plants had a lower osmotic potential, we determined the proline content of WT and transgenic plants. Drought stress induced a significant increase of proline content in both WT and transgenic tobacco lines, but the transgenic plants accumulated more proline compared with WT (**Figure 4D**).

### Transgenic Plants Accumulated Less ROS than WT under Water Stress

**FIGURE 5:**
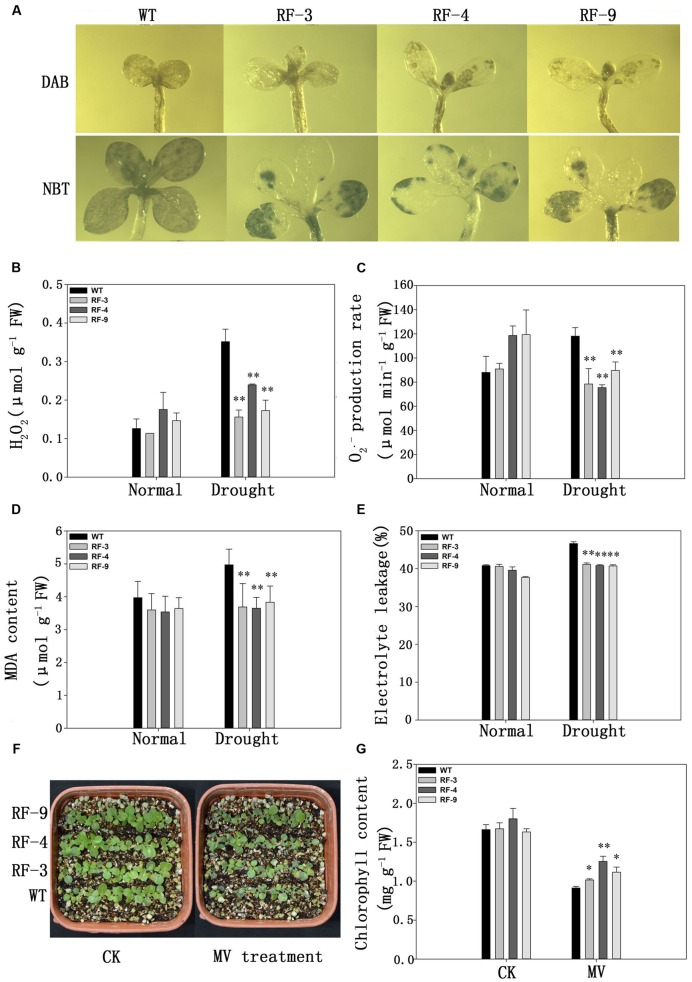
**Analysis of ROS accumulation and oxidative damage in WT and transgenic plants under water stress. (A)** *In situ* detection of H_2_O_2_ and O_2_^•-^ by DAB and NBT staining of WT and transgenic seedlings grown on normal medium for a week and then treated with 200 mM mannitol for 10 days. **(B–E)** H_2_O_2_ content, O_2_^•-^ production rate, MDA content and the electrolyte leakage of 60-day-old WT and transgenic plants treated with 20% PEG6000 for 3 days. **(F)** Phenotype of 2-week-old seedlings of MV treatments for 24 h. **(G)** Chlorophyll content of the seedlings in **(F)**. Each column represents the average of at least three replicates ± standard error (SE). “^∗^” and “^∗∗^” indicates significant differences compared with WT at *P* < 0.05 and *P* < 0.01.

Drought can induce the production and accumulation of ROS, which are toxic to plant cells. So we examined the levels of endogenous hydrogen peroxide (H_2_O_2_) and superoxide radical (O_2_^•-^) by DAB and NBT) staining. The WT plant leaves were more strongly stained with DAB and NBT than transgenic plant leaves under water stress (**Figure 5A**).

We also measured the H_2_O_2_ content and O_2_^•-^ production rates in the leaves. As the results of DAB and NBT staining assays, drought stress induced the accumulation of H_2_O_2_ levels and O_2_^•-^ production in the plant leaves examined. However, the levels of H_2_O_2_ andO_2_^•-^ were nearly 2- and 1.5-fold higher in WT plant, respectively, compared with those in the transgenic plants (**Figures 5B,C**).

Under drought conditions, plenty of ROS is produced in plant, which can induce membrane lipid peroxidation leading to an increase of the membrane permeability (Farooq et al., 2009). Then, we measured the electrolyte leakage of the transgenic plants under drought conditions. No significant difference was found between transgenic and WT plants without drought stress, but drought stress induced higher electrolyte leakage in WT plants compared with the transgenic lines (**Figure 5E**).

MDA content is usually considered as an index of membrane lipid peroxidation. Drought stress enhanced MDA contents of both transgenic and WT plants, whereas the MDA content was significantly lower in transgenic plants compared with WT plants under drought stress (**Figure 5D**).

Methylviologen (MV) can induce plant cells to produce excessive superoxide anions, which have a toxic effect on plant cells. Therefore, we observed the effects of MV treatment on the phenotypes of both WT and transgenic plants. From **Figure 5F**, no difference was observed between WT and transgenic lines under normal water conditions, but when exposed to MV (10 μM), WT plants exhibited a severely inhibited phenotype, while the transgenic plants displayed a higher tolerance to MV (**Figure 5F**), and this is further proved by the chlorophyll contents (**Figure 5G**). The results in **Figure 5** suggested that *TaFBA1* expression can decrease the ROS accumulation to protect tobacco from oxidative damage, and transgenic plants were less damaged by MV compared with WT.

### The Transgenic Plants have High Antioxidant Enzyme Activity

**FIGURE 6:**
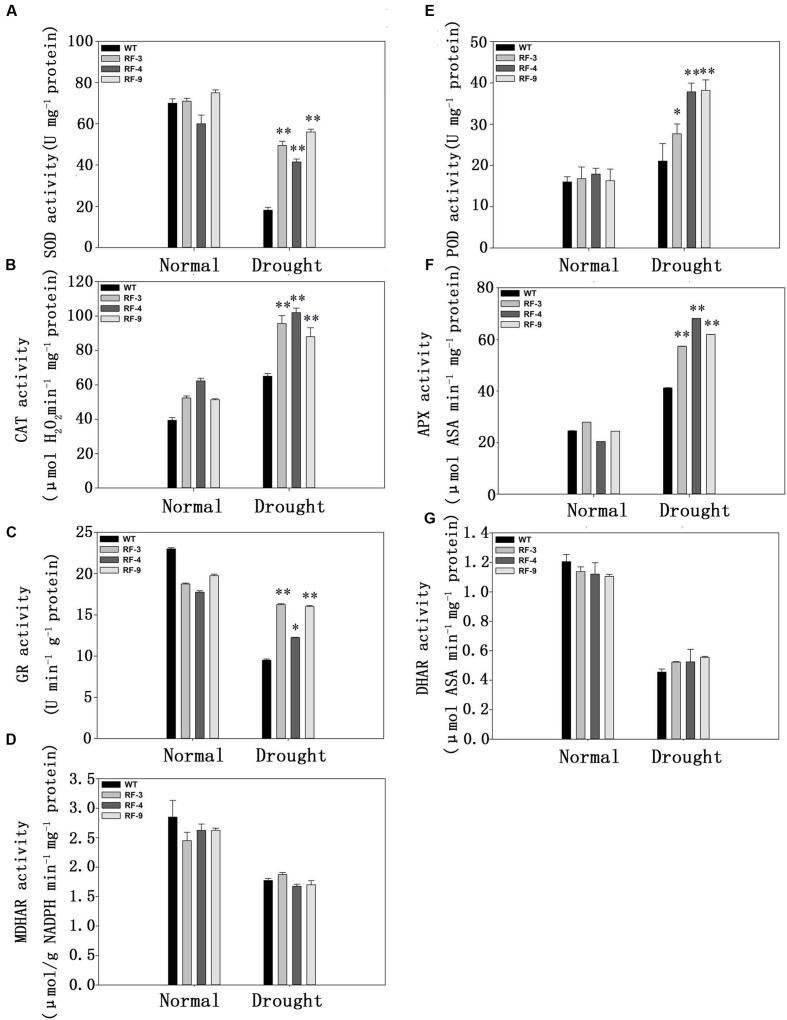
**The activities of the antioxidant enzymes in WT and transgenic plants under drought stress. (A)** SOD, superoxidase dismutase; **(B)** POD, guaiacol peroxidase; **(C)** CAT, catalase; **(D)** APX, ascorbate peroxidase; **(E)** GR, glutathione reductase; **(F)** DHAR, dehydroascorbate reductase; **(G)** MDHAR, monodehydroascorbate reductase. Each column represents the average of at least three replicates ± standard error (SE). “^∗^” and “^∗∗^” indicates significant differences compared with WT at *P* < 0.05 and *P* < 0.01.

As less ROS was detected in transgenic plants (**Figure 5**), we measured the activities of some antioxidant enzymes, which can reduce the ROS accumulation in plants. The data in **Figure 6** indicated that the activities of POD, CAT and APX were all significantly increased when treated with drought stress, but the activities of them in transgenic plants were higher than WT. As an exception, the activity of SOD was decreased when suffered to drought stress. However, the SOD activity was still much higher in transgenic plants than that of WT, which was similar to POD, CAT, and APX (**Figure 6A**).

Glutathione reductase, DHAR, and MDHAR are key enzymes of the ASA-GSH cycle, which are related to eliminate H_2_O_2_ and O_2_^•-^ (Noctor and Foyer, 1998). We next measured the activities of them. Under drought condition, the GR activity of transgenic plants was higher by about 1.5-fold than that of WT, while the activities of DHAR and MDHAR had no obvious difference between WT and transgenic plants under drought conditions.

Together, the results in **Figures 5** and **6** suggested that the enhanced tolerance to oxidative stress in transgenic plants may be due to the increase of antioxidant enzyme activity.

### *TaFBA1* Overexpression Induced the Expression of Some Antioxidant-Related Genes

**FIGURE 7:**
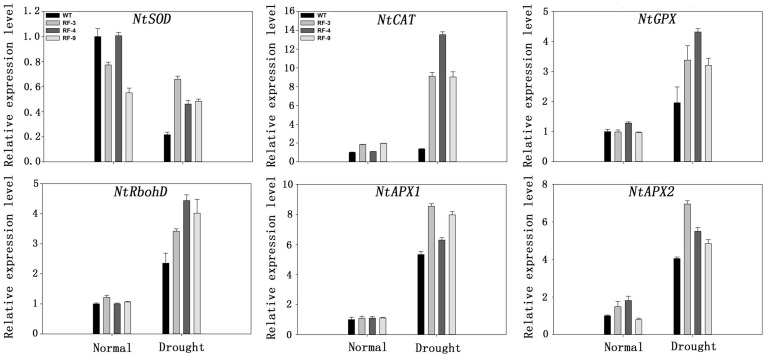
**Expression of antioxidant related genes in WT and transgenic plants under drought stress by real-time RT-PCR.** Transcript levels of these genes in transgenic plants are indicated relative to the levels in WT plants (set at 1), referring to the transcript of *actin* in the same samples. Each column represents the average of at least three replicates ± standard error (SE).

To investigate the mechanisms of the oxidant tolerance in transgenic plants during drought conditions, gene expression levels were analyzed quantitatively after the tobacco plants had been subjected to drought stress for a week. We analyzed the expression of *NtSOD, NtCAT, NtGPX, NtRbhoD, NtAPX1, NtAPX2*, which have been reported to enhance the oxidant tolerance in tobacco (Slooten et al., 1995). As shown in **Figure 7**, drought treatment up-regulated the expression of some antioxidant- related genes, including *NtCAT, NtGPX, NtRbhoD, NtAPX1, NtAPX2*, whereas the expression of *NtSOD* was decreased after drought stress treatment. Above all, the expression of these genes in transgenic plants was all higher than that in WT plants, which was similar to the change of antioxidant enzyme activity (**Figure 6**).

## Discussion

### Expressing *TaFBA1* Driven by *RD29A* instead of *35S* Promoter Eliminates its Impact on the Phenotype of Transgenic Plants

Promoter plays a decisive role in the gene expression. Transgenic plants are usually created with a functional gene under the control of the constitutive *CaMV 35S* promoter. The constitutive promoter leads to the expression of target genes in all tissues and organs of plants at all developmental stages, which usually influences the growth and development of transgenic plants (Jaglo-Ottosen et al., 1998; Gilmour et al., 2000). So the novel promoter that directs gene expression only in relevant stress is essential. In previous studies, the use of *RD29A* promoter showed more benefits than that of *CaMV 35S* (Yamaguchi-Shinozaki and Shinozaki, 1993; Kasuga et al., 2004; Pellegrineschi et al., 2004). *RD29A* contains dehydration responsive element (DRE) and can be stress-inducible. It allows low expression levels of target genes under normal conditions and a rapid increase of the genes when suffering dehydration stress (Yamaguchi-Shinozaki and Shinozaki, 1994). In our previous study, *TaFBA1* gene controlled by *35S* promoter was transformed into tobacco and some transgenic plants were obtained (Zhou et al., 2014). But we found that the phenotype of transgenic plants was much different from that of WT under normal water conditions (**Figure 1A**). To eliminate this diversity, stress-inducible promoter was used to generate transgenic tobacco plants with overexpression of *TaFBA1* (**Figure 1B**). Under normal condition without stress, the transformed gene was no expression, but drought stress induced its mRNA and TaFBA1 accumulation rapidly (**Figures 1C,D**). We found that the growth and developmental patterns of the *RD29A* transgenic plants were similar to that of WT under normal conditions (**Figure 1A**). In the following experiments, the *RD29A* transgenic tobacco lines, RF-3, RF-4, and RF-9 were used to examine their drought stress tolerance.

### Drought-Inducible Expression of *TaFBA1* Confers Drought Tolerance in Transgenic Plants

For most plants, seed germination and early seedling growth are highly sensitive to the abiotic stresses (Mito et al., 2010). Previous studies have also revealed that F-box genes play important roles in seed germination (Jia et al., 2011; Rajjou et al., 2012; Li Y.Z. et al., 2014). In **Figure 2**, under normal condition, no significant difference in germination and seedling growth was found between the transgenic lines and WT. But the transgenic lines showed a better germination and growth, and a higher survival rate than WT under drought stress. When exposed to drought conditions, the phenotype and physiological observations of the grown transgenic plants were also superior compared with these of WT (**Figures 3A,B**).

Stomata is one of the first induction in plants to drought stress, and drought can lead to the closure of stomata, which can limit the gas exchange between the cell and atmosphere and will cause the reduction in photosynthesis (Farooq et al., 2009; Bhargava and Sawant, 2013). Drought stress can also influence the stability of thylakoid membrane to depress the photosynthesis of plants (Chaves et al., 2009). The Pn of transgenic plants was higher than WT when suffering drought stress (**Figure 3C**). This may related to the higher chlorophyll content in transgenic plants (**Figure 3D**).

### Transgenic Plants Remain High Water Content by Decreasing of the Osmotic Potential

Drought is one of the most common factors of abiotic stresses, which is usually due to the continuous water loss through transpiration and evaporation into atmosphere and less water taken from the soil (Bhargava and Sawant, 2013; Koﬄer et al., 2014). Drought resistance can be due to the drought avoidance via more water retention and less water loss. From **Figures 4A,B**, the transgenic tobacco plants have a high water content and low water loss.

Osmotic regulation ability also participates in the water conservation. When plants are exposed to drought stress, they actively accumulate solutes and, as a result, ψ_s_ drop, promoting the flow of water into the cell (Chaves et al., 2009). Proline, as a common solute, can be used as index of osmotic adjustment in plants. From **Figure 4D**, the proline contents were higher in transgenic plants than that of WT when suffering drought stress, which may contribute to the low osmotic potential in transgenic lines (**Figure 4C**). All these data suggest that expression of *TaFBA1* confers drought tolerance in transgenic tobacco. Transgenic plants remain high water content may be by decreasing of the osmotic potential via accumulating of some solutes such as proline.

### The Expression of *TaFBA1* Results Low ROS Accumulation under Drought Conditions

Drought stress usually results in stomatal closure. The stomatal closure caused by drought stress limits CO_2_ uptake by leaves, leading to the exhaustion of the primary electron acceptor NADP and the block of the electron transport to NADP, which contributes to the formation of relative oxygen species (ROS; Foyer and Noctor, 2009; Miller et al., 2010; Koﬄer et al., 2014). Excess production of ROS destroys the cellular structures and the metabolism in plants (Bartels and Sunkar, 2005). The *TaFBA1* transgenic plants accumulated less ROS contents than WT under drought condition (**Figures 5A–C**).

The cell membrane is the main target in the process of oxidative damage induced by drought stress. Therefore, cell membrane stability is commonly used as an index of stress tolerance (Yue et al., 2011). MDA is the final product of lipid peroxidation and is usually used as an index of the level of membrane damage (Moore and Roberts, 1998). Electrolyte leakage, MDA content in both transgenic and WT plants increased under drought stress, while those of transgenic plants were lower than WT (**Figures 5D,E**), suggesting that the transgenic plants have a weaker membrane damage caused by ROS than WT under drought stress.

As a secondary stress, oxidative stress is extensive in most abiotic stresses (Dinakar et al., 2012). Plants own antioxidant systems keeping the ROS at a steady level to protect plants from oxidant damage. Some antioxidant enzymes, such as SOD, POD, CAT and APX, are involved in this process. The activities of all the antioxidant enzymes in the transgenic plants were higher significantly compared to that of WT under drought stress (**Figures 6A–D**). The higher mRNA levels of antioxidant genes in transgenic plants under drought stress may be involved in the enhanced activities of antioxidant enzymes in transgenic plants (**Figure 7**).

The ascorbate–glutathione (ASA–GSH) cycle, a non-enzymatic antioxidant defense systems, is also involved in removing the excess of ROS. GR, DHAR and MDHAR, key enzymes of the cycle, play an important role in the non-enzymatic system (Hoque et al., 2007). GR, DHAR, and MDHAR activities were higher in transgenic plants than in the WT (**Figures 6E–G**), suggesting the possible involvement of glutathione cycle in the defense of oxidant damage. All these revealed that plants can protect themselves from oxidant stress caused by drought stress by both enzymatic and non-enzymatic antioxidant defense systems.

## Conclusion

Our results suggest that the expression of *TaFBA1* driven by *RD29A* promoter in tobacco can confers enhanced tolerance to drought stress, probably first by keeping high water retention and low osmotic potential, then by scavenging the excrescent ROS and decreasing the oxidative damage, while no significant difference in phenotype between WT and transgenic plants under normal water conditions. Meanwhile, our results will be useful to elucidate the function of the F-box gene in plant abiotic stress responses.

## Author Contributions

Conceived and designed the experiments: SZ, WW. Performed the experiments: XK, SZ, SY, ZZ. Analyzed the data: XK, SZ, YH. Contributed reagents/materials/analysis tools: SZ, WW. Wrote the paper: XK, SZ. Proof read and final approval: SZ, WW.

## Conflict of Interest Statement

The authors declare that the research was conducted in the absence of any commercial or financial relationships that could be construed as a potential conflict of interest.
